# Problem-solving therapy for suicide prevention outcomes in the VA’s suicide prevention 2.0 clinical telehealth program

**DOI:** 10.1007/s10865-026-00651-9

**Published:** 2026-04-29

**Authors:** Jessica A. Walker, Julie Lutz, Lisanne van Engelen, Sherry A. Beaudreau, Kelly L. Green, Viviana Padilla-Martinez, Gregory K. Brown, Angelic D. Chaison, Jennifer F. George, Kathleen N. Rekart, Shannon Sisco, Julie Loebach Wetherell, Nicole Bekman, Rani Hoff, Meaghan Stacy

**Affiliations:** 1 Office of Suicide Prevention, Veterans Health Administration, Washington, DC USA; 2 Sierra Pacific Mental Illness Research, Education, and Clinical Center (MIRECC), VA Palo Alto Health Care System, Palo Alto, CA USA; 3 Department of Veterans Affairs, Northeast Program Evaluation Center (NEPEC), West Haven, CT USA; 4 Department of Psychiatry and Behavioral Sciences, Stanford University School of Medicine, Stanford, CA USA; 5 Department of Psychiatry, Perelman School of Medicine, University of Pennsylvania, Philadelphia, PA USA; 6 Bay Pines VA Healthcare System, Bay Pines, FL USA; 7 Office of Mental Health (OMH), Veterans Health Administration, Washington, DC USA; 8 Department of Psychiatry and Behavioral Sciences, Baylor College of Medicine, Houston, TX USA; 9 Manchester, New Hampshire VA Medical Center, Manchester, NH USA; 10 Michael E. DeBakey VA Medical Center, Houston, TX USA; 11 VA San Diego Healthcare System, San Diego, CA USA; 12 Department of Psychiatry, University of California, San Diego, San Diego, CA USA; 13 Department of Psychiatry, Yale University School of Medicine, New Haven, CT USA

**Keywords:** Veterans, Suicidal ideation, Suicidal behavior, Cognitive behavioral therapy, Evidence-based practice, Depression

## Abstract

U.S. Veterans are at elevated risk for suicide, and suicide prevention is a top clinical priority of the Department of Veterans Affairs (VA). Problem-Solving Therapy for Suicide Prevention (PST-SP) is an evidence-based practice offered in the VA’s national Suicide Prevention (SP 2.0) Clinical Telehealth Program, through a fully virtual model. This paper presents clinical outcomes from this program. PST-SP was delivered to 3754 VA-enrolled Veterans with suicidal self-directed violent behaviors in the past year, and 2269 completed a full course of PST-SP. Veterans completed a median of 7 and mode of 6 sessions of PST-SP. Suicide-related coping, suicide-relevant cognitions, negative problem-solving beliefs, and depressive symptoms significantly improved throughout treatment. Results indicated that providing PST-SP treatment via telehealth is feasible and acceptable to Veterans enrolled in VA with a recent history of suicidal self-directed violence behaviors. Veterans demonstrated clinical improvement in suicide and mental health-related outcomes with PST-SP treatment.

## Introduction

Suicide among United States (U.S.) Veterans is a critical national priority. Veterans are at significantly elevated risk of suicide. In 2023, 6398 Veterans died by suicide, an average of 17.5 Veteran suicides per day (U.S. Department of Veterans Affairs Office of Suicide Prevention [VA OSP], 2025). Veterans’ rates of suicide are twice as high as the general U.S. population, at 35.2 per 100,000 Veterans, compared with 16.9 per 100,000 in the general population (VA OSP, 2025). The U.S. Department of Veterans Affairs (VA) identified Veteran suicide prevention as the top clinical priority in its 2018–2024 Strategic Plan (U.S. Department of Veterans Affairs, 2018), and outlined a ten-year National Strategy for Preventing Veteran Suicide that includes promoting treatments for Veterans at risk for suicide and evaluating the impact and effectiveness of such interventions (U.S. Department of Veterans Affairs Office of Mental Health and Suicide Prevention, 2018).

A key component of suicide prevention in the VA and other healthcare systems is the implementation of evidence-based practices (EBPs) to address risk factors and reduce suicide risk (U.S. Department of Veterans Affairs & Department of Defense [VA/DoD], 2024). Systematic reviews of EBPs for suicide prevention (EBP-SPs) have examined suicide prevention practices in multiple domains including pharmacotherapy (e.g., antidepressants), psychotherapy (e.g., cognitive behavioral therapy, dialectical behavior therapy), and other interventions (e.g., lethal means safety). However, evidence is generally weak or insufficient for most practices, including psychotherapies, and more data are needed to establish the efficacy of these practices (Mann et al., 2021; Zalsman et al., 2016). The VA and Department of Defense (DoD) have released Clinical Practice Guidelines for Assessment and Management of Patients at Risk for Suicide (CPGs) since 2019, with the most recent update in 2024, based on a rigorous process of evidence review (VA/DoD, 2024). The guidelines include recommendations for evidence-based treatment of Veterans at appropriate levels of risk (e.g., lower acute but higher chronic risk might benefit from psychotherapy such as cognitive behavioral therapy or problem-solving therapy).

A critical component in Veteran suicide prevention is not merely the existence of evidence-based practices for suicide prevention (EBP-SPs) but also access to such interventions. Synchronous telemental health makes treatment more widely available for individuals with barriers to in-person VA treatment, especially with flexible implementation and considerations for personalization (Lindsay et al., 2019). Telemental health care can be used to effectively manage mental health (Sugarman & Busch, 2023; Watanabe et al., 2023) and could be especially useful for management of suicide risk in individuals in rural areas where access to treatment may otherwise be very limited (Rojas et al., 2020).

Combining VA’s priorities of suicide prevention and Veterans’ need for scalable, accessible care, the VA established the national Suicide Prevention (SP 2.0) Clinical Telehealth Program to provide EBP-SPs via telemental health care. Telehealth treatment is offered to Veterans nationwide with a recent history of suicidal self-directed violence (SSDV). SP 2.0 Clinical Telehealth represents the first and only nationwide, fully virtual, evidence-based treatment program for Veterans with a recent history of suicidal self-directed violence (SSDV; Landes et al., 2026). Partnering with the VA’s Clinical Resource Hub infrastructure (Burnett et al., 2023), over 100 psychotherapists were hired and are embedded within 18 regional telemental health networks to provide virtual care to Veterans within every health care system in the nation. SP 2.0 therapists are trained in multiple EBP-SPs recommended by the VA/DoD CPGs (VA/DoD 2019, 2024). Problem-Solving Therapy (PST) is one non-pharmacologic treatment recommended in the current guidelines for reducing suicidal ideation (SI; VA/DoD, 2024) and is one of four interventions offered in SP 2.0.

The effect of PST on suicide risk is theorized to be through improvement of problem-solving skills, which are impaired among those who attempt suicide (Dieserud et al., 2003; Gibbs et al., 2009; Schotte & Clum, 1987). PST is a flexible strengths-based psychotherapy that reduces stigma by framing coping with stressors and problems as normal, and therapists teach clients to build concrete tools and strategies with the overall goal of improving problem-solving for diverse life problems (Nezu & Nezu, 2019). PST is considered part of the cognitive-behavioral therapy (CBT) family of treatments but tends to be more concretely structured around clients’ day-to-day problems compared with more traditional cognitive restructuring-focused CBT. PST protocols have been developed and effectively implemented within various settings in VA, such as for managing wellness of transitioning service members (Tenhula et al., 2014); mental health and stress in primary care, home-based primary care and community living centers (VA nursing homes; Beaudreau et al., 2022a, 2022b; Funderburk et al., 2021; Wetherell et al., 2023); and web-based self-management courses (U.S. Department of Veterans Affairs, 2020). Though to date there are no meta-analyses of PST for suicide prevention, meta-analyses of PST for depression in adults indicate effectiveness over control treatments, with comparable effects to other psychological treatments and potentially longer-lasting effects compared to other depression treatments (Cuijpers et al., 2018, 2021).

Though PST has been identified as a treatment with strong potential for reducing SI and behaviors, additional data are necessary in this area. A pilot of PST for suicide prevention in Veterans with moderate-to-severe traumatic brain injury (TBI) found that PST was acceptable and feasible in this population (Barnes et al., 2017). In a randomized controlled trial (RCT) of PST for individuals presenting to the hospital following self-harm, PST reduced chances of presenting again among individuals with a history of repeated self-harm (as opposed to first-time episode of self-harm; Hatcher et al., 2011). Initial case reports and secondary analyses of clinical trials focusing on PST for other problems (e.g., depression, anxiety) have indicated reductions in SI (Choi et al., 2016; Gustavson et al., 2016; Hopko et al., 2013; Lutz et al., 2020, 2021). To further establish the efficacy of PST for suicide prevention, more rigorous RCTs are needed. Some such trials are underway, including among older Veterans with SI at the VA (Beaudreau et al., 2023), and among men presenting to the emergency department with self-harm (Hatcher et al., 2020, 2022). In this paper, we present clinical outcomes from PST in SP 2.0, including characteristics of Veterans who receive PST treatment, initial suicide-related outcomes, and longer-term SSDV outcomes.

## Methods

### SP 2.0 clinical telehealth program

Veterans are referred after a recent (past 12 months) SSDV (i.e., suicide attempt or preparatory behavior), most frequently from outpatient general mental health and from Suicide Prevention Coordinators. Veterans complete an SP 2.0 intake session. Therapists and Veterans collaboratively decide on a treatment plan using shared decision-making, and PST is one of the treatment options discussed and offered. For further details of the SP 2.0 program, see Landes et al. (2026). The program was opened to Veterans in April 2021, standardized health record documentation was implemented October 2021, and by September 30, 2025, the program processed 33,190 referrals and completed 17,504 intake appointments.

### PST for Suicide Prevention (PST-SP) treatment

The PST protocol for SP 2.0 (PST-SP) was adapted by experts in PST and suicide prevention treatment, based upon the emotion-centered PST framework of Nezu and Nezu (2019). Emotion-centered PST includes tools to further facilitate better problem-solving by reducing cognitive overload, managing emotional distress, and increasing motivation and hope, in addition to the structured problem-solving steps included in traditional PST. PST-SP was designed to use a case formulation approach to address problems most proximally related to an individual Veteran’s suicide risk. Veterans are provided handouts to reinforce learning and engage in experiential practice during and outside of session. Veterans receive the Stanley-Brown Safety Planning Intervention (SPI: Stanley & Brown, 2012) for managing acute suicidal crises prior to beginning PST-SP, and their safety plan is reviewed and revised as indicated during treatment. Ongoing suicide risk assessment is also conducted at the beginning of every session to monitor and respond to changes in risk over the course of treatment. Generally, the first PST-SP session provides an introduction and rationale, subsequent sessions focus on the delivery of four toolkits—managing emotional distress, increasing motivation and hope, reducing cognitive overload, and structuring problem-solving steps—and the final session involves skill review and relapse prevention. The PST-SP protocol clearly delineates when the Veteran should use problem-solving skills (i.e., for problems that can lead to suicidal thinking or behaviors) and when they should use the safety plan (i.e., for managing an acute suicidal crisis). Treatment was intended to be delivered in 6 to 12 sessions. Clinicians delivering PST-SP in SP 2.0 successfully completed a rigorous training program for PST-SP that included (1) individual independent learning, (2) a live group-workshop with didactics and role play practice, and (3) five months of weekly consultation, during which clinicians had to demonstrate competent delivery of PST-SP on at least two completed cases based on audio review of sessions by a PST-SP consultant.

### Measures and variables

PST-SP session data were collected through (1) VA Electronic Health Record (EHR) note templates submitted between October 21, 2021 and September 30, 2025, and (2) EHR data on demographic and clinical characteristics including age, birth sex, race, ethnicity, and marital status. Mental health and medical diagnoses (major depressive disorder, any substance use disorder, bipolar disorder, posttraumatic stress disorder [PTSD], traumatic brain injury [TBI], any pain disorder) were recorded for Veterans with at least one outpatient encounter or bed stay with the diagnosis (or two outpatient encounters or one bed stay for PTSD). The Elixhauser Comorbidity Index (Elixhauser, et al., 1998) comprised a count of the number of serious medical conditions noted in the EHR. Baseline acute and chronic suicide risk levels consistent with the CPGs (low, intermediate, or high; VA/DoD, 2024) were gathered from the SP 2.0 intake.

Treatment outcomes as assessed as part of VA measurement-based care (Resnick & Hoff, 2020) in the PST-SP program included the following four measures. The *Suicide-Related Coping Scale* (SRCS; Stanley et al., 2017) is a 17-item self-report measure of knowledge and self-efficacy in using internal and external coping strategies to manage suicidal thoughts, urges, and crises. Items are rated 0 (Strongly disagree) to 4 (Strongly agree), yielding a total score of 0 to 68, with higher scores indicating better suicide-related coping. No timeframe is explicitly stated, thus respondents rate items as they currently feel. The *Suicide Cognitions Scale—Revised* (SCS-R; Bryan et al., 2021) is a 16-item self-report measure of suicide-relevant thoughts (e.g., hopelessness, burdensomeness) rated 0 (strongly disagree) to 4 (strongly agree) on how they feel “right now,” for a total score of 0 to 64, with higher scores indicating greater suicide-relevant cognitions. The *Patient Health Questionnaire-9* (PHQ-9; Kroenke et al., 2001) is a 9-item self-report measure of depressive symptoms experienced in the past 2 weeks, rated 0 (not at all) to 3 (nearly every day) for a total score of 0 to 27. Though clinical cut-offs were not used for the purposes of the current analyses, the PHQ-9 scores of 5–9 (mild), 10–14 (moderate), 15–19 (moderately severe), and 20–27 (severe) indicate clinically significant depression. Finally, the *Negative Problem Orientation Questionnaire* (NPO-Q; Robichaud & Dugas, 2005) is a 12-item self-report measure of negative beliefs about problems, rated 1 (not at all true of me) to 5 (extremely true of me) for a total score of 12 to 60, with higher scores indicating a greater negative orientation toward problems. No timeframe is explicitly stated, thus respondents rate items as they currently feel.

Other indices of treatment outcome included PST-SP discharge type and SSDV. A discharge note was completed for every Veteran at discontinuation of PST-SP. The discharge note indicates partial or full completion of the PST-SP protocol. SSDV was recorded in the VA EHR via Comprehensive Suicide Risk Evaluations (CSREs); Suicide Behavior and Overdose Reports (SBORs); and/or inpatient, emergency department, or urgent care visits/admissions for suicide behaviors.

### Analyses

Complete administrative data were available from 133 of 139 healthcare systems due to a transition to a new federal EHR during the project period, with data on n = 4950 Veterans who had at least one PST-SP session with a SP 2.0 provider. Veterans were included in analyses only if they had complete demographic data, intake note data, a subsequent PST-SP session note, and had been discharged from PST-SP (i.e., not still in process), excluding n = 1196 Veterans. Descriptive statistics are presented for demographics, clinical characteristics, and baseline/intake measures for all included Veterans. We created a focused sample of Veterans who received a full episode of care consistent with the intentions of the PST-SP treatment protocol—this included Veterans who received between 5 and 12 sessions (Veterans with 5 sessions have the opportunity to receive all PST toolkits, and PST-SP is designed to be delivered in up to 12 sessions), within up to 4 months (PST-SP is meant to be delivered weekly, and 4 months allows for up to 12 sessions weekly with occasional gaps). This focused sample further excluded 1485 Veterans. Using this sample, we analyzed the four outcome measures (SRCS, PHQ-9, NPO-Q, and SCS-R) by generating generalized linear models. Pairwise deletion was used for each analysis, such that Veterans with missing data on the variables in any individual analysis were excluded. Data were included in analyses if the measure was completed at baseline/first session and within 3 days of the final session. Measures were also often administered throughout the treatment phase. To create these models, we first determined the base model of best fit for each outcome based on baseline score, time, the interaction of time with time (time * time) to test possible curvilinear/quadratic patterns, and the interaction between baseline score and time (baseline * time). Once we selected the base models, we added demographic and clinical variables in a stepwise manner to examine incremental significance in the model. Alpha was set at α < 0.003 for significance of results, adjusting for multiple comparisons. The final models included all significant variables; four demographic variables were included in all models—sex, age, ethnicity, race. To generate visualizations of the final model results, estimates at low (one standard deviation below the mean), average (mean), and high (one standard deviation above the mean) were calculated and graphed.

## Results

### Program and veteran characteristics

**Fig. 1:**
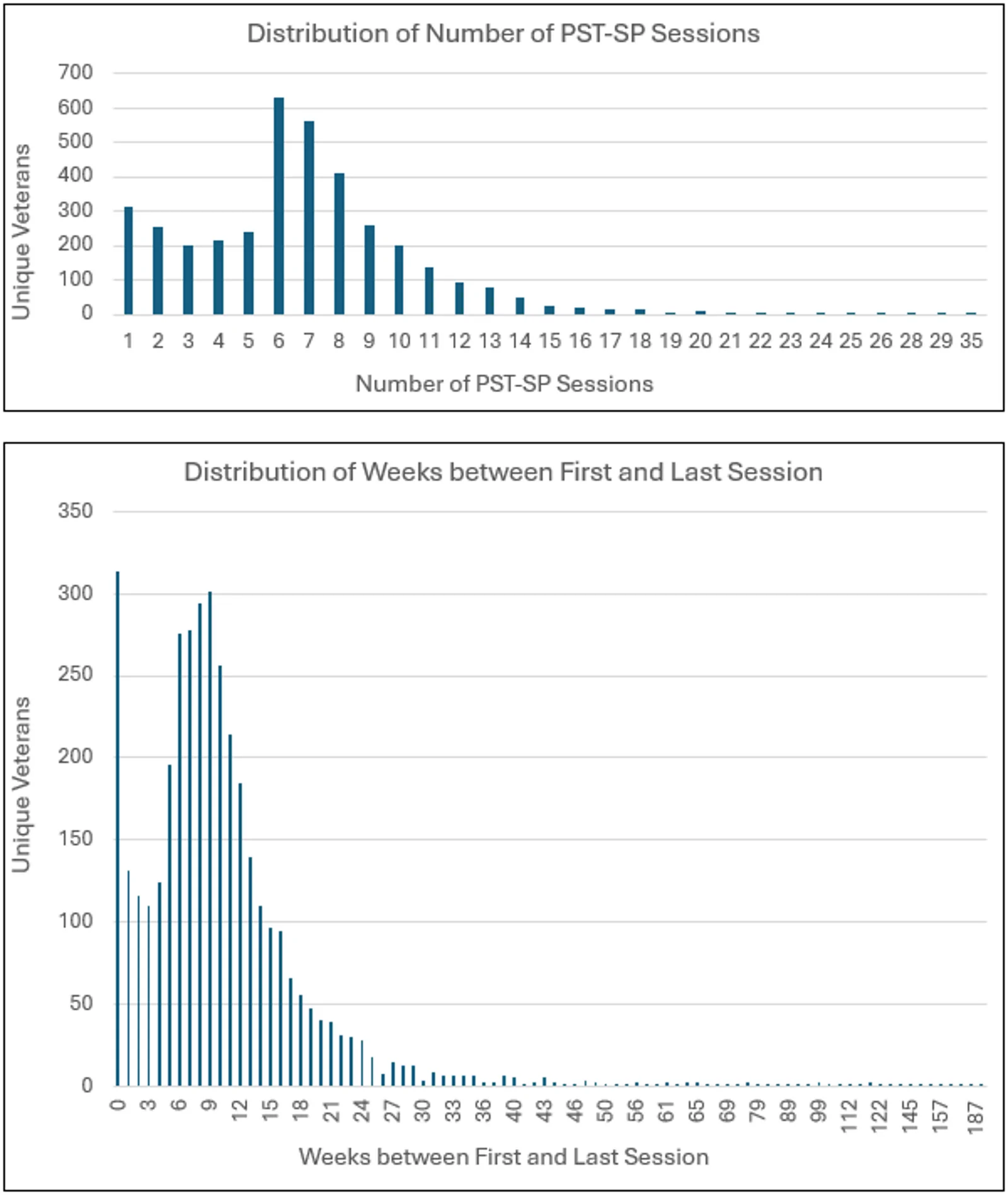
Distributions of number of PST-SP sessions and weeks between first and last session

A total of 25,203 documented PST-SP therapy sessions exist for 3754 unique Veterans in the full PST-SP population. Characteristics of the full PST-SP population and focused episode of care sample (total n = 2269) are provided in Table 1. See Fig. 1 for the distribution of number of sessions and number of weeks between first and last session in the full PST-SP population. Veterans in the full PST-SP population had a median of 7 sessions and a mode of 6 sessions, with the total number of PST-SP sessions ranging from 1 to 35. Median time between the first and last session was 9 weeks, with a range from 0 to 187 weeks. Of the Veterans with 0 weeks, most (n = 312) of the Veterans had a total of one session. Most (86.46%) PST-SP sessions were Video to Veteran residence and only 5.76% of sessions reported any connectivity issue disrupting video. For Veterans in the focused episode of care sample, the median number of sessions continued to be 7 with a mode of 6. Median and mode number of weeks between the first and last session was 9 weeks, with a range of 2 to 18 weeks. In the full PST-SP population, 64.81% indicated a full protocol was completed. For the focused episode of care sample, 85.41% indicated a full protocol.

**Table 1:** Characteristics of PST-SP Veterans compared to VA population with mental health diagnoses

Characteristics at Intake (PST-SP Veterans) or Time of Data Extraction (VA Population)	Veterans with Mental Health Diagnoses Receiving VA Care Nationally, Oct 1 2024–Sep 30 2025	Veterans Enrolled in PST-SP Program % (n = 3754)	Veterans Who Received Full Episode of PST-SP % (n = 2269)
Female sex	16.17%	22.88% (859)	22.39% (508)
Age in years—mean (SD)	55.97 (16.86)	43.15 (13.64)	43.71 (13.83)
Currently married	49.55%	43.85% (1646)	44.95% (1020)
Hispanic ethnicity	9.95%	12.65% (475)	11.99% (272)
Race—American Indian or Alaska Native	1.39%	1.52% (57)	1.37% (31)
Asian	1.92%	3.20% (120)	3.39% (77)
Black or African American	21.54%	21.87% (821)	21.42% (486)
Native Hawaiian or Other Pacific Islander	1.35%	2.10% (79)	1.81% (41)
White	66.36%	62.55% (2348)	63.33% (1437)
Unknown	8.96%	8.58% (322)	8.86% (201)
Rurality—Highly rural	0.92%	0.61% (23)	0.66% (15)
Rural	31.00%	26.77% (1005)	26.66% (605)
Urban	67.61%	68.57% (2574)	68.97% (1565)
Missing data	0.47%	4.05% (152)	3.70% (84)
Homeless	3.22%	9.22% (346)	8.55% (194)
Any Mental Health Diagnosis	100.00%	99.09% (3720)	99.03% (2247)
Major Depressive Disorder (MDD)	34.13%	81.86% (3073)	81.27% (1844)
Substance Use Disorder	22.68%	42.41% (1592)	40.50% (919)
Serious Mental Illness	8.45%	20.67% (776)	20.93% (475)
Bipolar Disorder	5.44%	16.14% (606)	16.22% (368)
Personality Disorder	2.14%	18.17% (682)	17.32% (393)
Posttraumatic Stress Disorder (PTSD)	34.45%	66.44% (2494)	66.06% (1499)
Possible Traumatic Brain Injury	4.25%	9.51% (357)	9.78% (222)
Dementia Diagnosis	3.18%	0.80% (30)	1.10% (25)
Any Pain Diagnosis	72.93%	72.14% (2708)	72.68% (1649)
Elixhauser Comorbidity Index—mean (SD, range)	2.98 (2.51, 0–23)	3.10 (2.16, 0–18)	3.13 (2.20, 0–18)
Acute Suicide Risk—High	N/A	0.24% (9)	0.13% (3)
Intermediate	N/A	28.21% (1059)	29.09% (660)
Low	N/A	22.16% (832)	21.86% (496)
Unknown	N/A	49.39% (1854)	48.92% (1110)
Chronic Suicide Risk—High	N/A	21.42% (804)	20.10% (456)
Intermediate	N/A	27.54% (1034)	29.40% (667)
Low	N/A	1.68% (63)	1.63% (37)
Unknown	N/A	49.39% (1854)	48.92% (1110)

Most PST-SP Veterans were male, White, living in an urban environment, and had a mental health and/or substance use disorder diagnosis. When compared with VA-wide Veterans with mental health diagnoses, the PST-SP program contained a higher proportion of females, Veterans with a Hispanic ethnicity, homeless Veterans, Veterans with TBI, and Veterans with any of the main psychiatric disorders that were tracked (i.e., likely comorbidity). Participants in the PST-SP program also tended to be younger. Characteristics of the larger PST-SP cohort and the focused sample were very similar.

### Short-term treatment outcomes

**Fig. 2:**
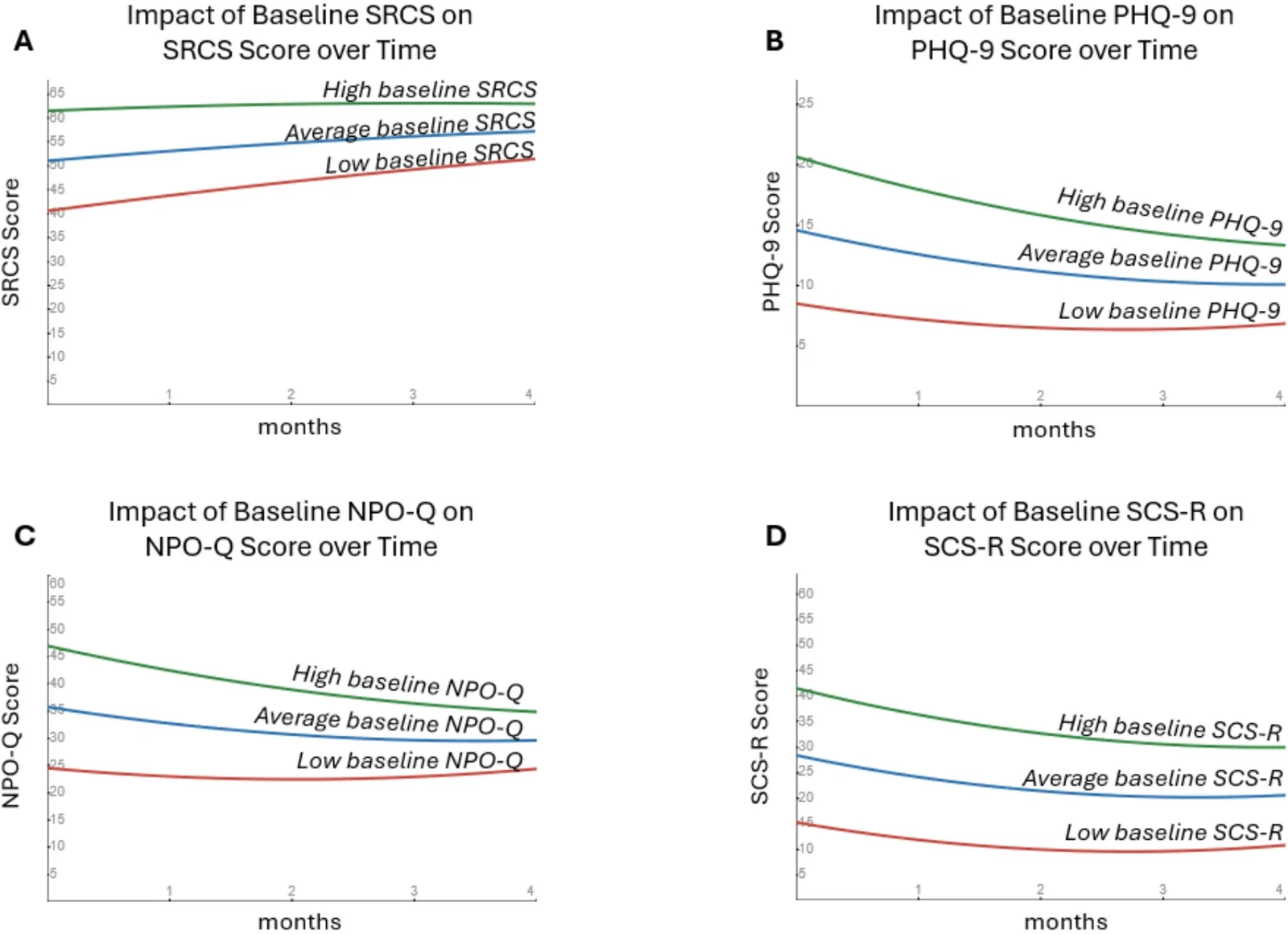
SRCS, PHQ-9, NPO-Q, and SCS-R short-term treatment outcome models

Table 2 displays the final model results predicting SRCS, PHQ-9, NPO-Q, and SCS-R scores. To understand the theoretical trajectories of Veterans’ scores over time, we created linear models (Fig. 2) showing score trajectories of Veterans with high (one standard deviation above mean), average, and low (one standard deviation below mean) baseline scores on each outcome measure. Due to smaller sample sizes and strict inclusion criteria for these models, we conducted sensitivity analyses with the full population, removing only outliers (i.e., received only the first introductory session of PST-SP which does not include any PST toolkits, or received more than 18 sessions; more than 6 months between first and last session). Results were similar in direction of trends, confirming that restriction of the focused sample did not artificially generate spurious effects.

**Table 2:** Results of generalized linear models predicting scores over time on suicide-related outcomes

	SRCS	SCS-R	PHQ-9	NPO-Q
N (surveys)	1738	7499	2268	2947
N (unique Veterans)	561	1361	741	1104
Baseline mean	51.27	28.02	14.88	35.79
Baseline SD	11.40	14.06	6.65	11.76
Low baseline (one SD below mean)	39.87	13.96	8.23	24.03
High baseline (one SD above mean)	62.67	42.08	21.53	47.55
Parameters				
Intercept	4.25***	3.89***	2.46***	3.73***
Time	7.47***	− 3.37***	− 0.52	1.25
Time × Time	− 0.17***	0.77***	0.29***	0.50***
Baseline	0.92***	0.93***	0.91***	0.95***
Baseline × Time	− 0.10***	− 0.06***	− 0.11***	− 0.13***
Sex	0.16	− 0.24	− 0.50	0.23
Age	− 0.01	− 0.02	− 0.03**	− 0.03
Ethnicity	1.15	0.40	0.13	0.11
Ethnicity × Time			− 0.61**	
Race	0.72	− 0.73	− 0.10	− 1.01**
SMI		− 1.85***		
Acute risk level			0.04	− 0.26
Acute risk level x Time			− 0.55**	− 0.97**

Results in table are from final models for each outcome. Only intercept, time (linear and quadratic), baseline, baseline * time interaction, critical demographic variables (i.e., sex, age, ethnicity, race) and significant other parameters of interest are shown (not all covariates shown, for ease of reading). If interaction is significant, parameters for main effect are also included. SMI = severe mental illness (includes psychotic and bipolar disorders)

**p < 0.003

***p < 0.001

#### SRCS

Time (linear and quadratic), baseline SRCS score, and the interaction between time and baseline score significantly predicted SCRS scores. All three (high, average, and low baseline) groups experienced an increase in scores (i.e. improvement in suicide-related coping) over time, with the low baseline score group showing the most improvement (increase of 6.10 points between baseline and midpoint) as compared to the average and high baseline score groups, who had an increase of 3.75 and 1.40 points, respectively.

#### SCS-R

Time (linear and quadratic), baseline score, and the interaction between time and baseline score significantly predicted SCS-R scores. Serious mental illness (SMI) diagnosis (including psychotic and bipolar disorders) was also significant. The three baseline groups all showed improvement in SCS-R score over time, but Veterans with a high baseline score showed the greatest decrease (reduction of 8.67 points at midpoint) while Veterans with a low baseline score showed the smallest decrease (reduction of 5.32 points at midpoint). Veterans with SMI started and ended PST-SP treatment with lower SCS-R scores than those without SMI.

#### PHQ-9

Time (quadratic), baseline PHQ-9 score, the interaction between time and baseline score, baseline age, the interaction between time and ethnicity, and the interaction between time and acute risk level significantly predicted PHQ-9 scores. All three baseline groups showed a decrease in PHQ-9 score over time. The high baseline score group showed the most improvement, with a decrease of 4.84 points at midpoint, as compared with the average baseline score group (decrease of 3.42 points) and the low baseline score group (decrease of 2.00 points). Baseline age had an effect on baseline PHQ-9, with the younger age group having a slightly higher starting score. Hispanic Veterans showed a larger decrease in PHQ-9 scores over time, with a decrease of 4.51 points at midpoint, as compared to 3.30 points for non-Hispanic Veterans. Finally, Veterans with a low acute risk level also saw a larger decrease in PHQ-9 score over time, with 4.26 points, as compared to 3.15 points for Veterans with a non-low acute risk level.

#### NPO-Q

In the linear model of NPO-Q scores, time (quadratic), baseline NPO-Q score, the interaction between time and baseline score, race, and the interaction between intake acute risk level and time were significant. All three baseline level groups showed a decrease in NPO-Q score over time, with the high baseline group showing the greatest improvement (a decrease of 8.06 points at midpoint). Compared to White Veterans, non-White Veterans started with higher scores (36.40 vs. 35.39). Finally, Veterans with a low intake acute risk level showed greater improvement over time, with a decrease of 6.55 points at midpoint as compared to 4.61 points for Veterans with a non-low intake acute risk level.

#### SSDV

Limiting our analysis to Veterans who had reached at least 18 months post-discharge, to allow sufficient time for observation of occurrences of SSDV, resulted in a sample of 1933 (51.49% of all Veterans in the full PST-SP population). In this sample, 22.92% (N = 443) had at least one recorded SSDV at any time post-discharge. Within 3-months post-discharge, 137 Veterans (7.09%) had SSDV, and 200 Veterans (10.35%) had SSDV within 6-months post-discharge. At 12 months post-discharge, the number of Veterans with SSDV was 308 (15.93%), and at 18 months, 369 Veterans (19.09%) presented with SSDV. For indirect comparison and context, we built a cohort of Veterans with an individual or group general mental health encounter during the same analysis dates (April 1, 2021 through September 30, 2025) who had SSDV in the year prior to their visit and had not received treatment through the SP 2.0 program; this cohort was not matched on other variables and therefore must not be construed as a direct equivalent comparison group. Of these 39,480 Veterans, 5613 (14.22%) had at least one recorded SSDV at any time post-encounter; 4.83% (N = 1907) had SSDV within 3 months post-encounter, 6.94% (N = 2738) within 6 months post-encounter, 10.11% (N = 3991) within 12 months post-encounter, and 12.31% (N = 4861) within 18 months post-encounter.

## Discussion

Central to addressing suicide prevention in Veterans—a top clinical priority—is Veterans’ access to EBP-SPs. The SP 2.0 Clinical Telehealth program was created to provide nationwide virtual access to EBP-SPs (recommended by CPGs) to a high-risk Veteran population defined as those with recent SSDV. PST-SP is offered as one treatment option, and after enterprise-wide dissemination, Veterans who received PST-SP demonstrated improvements in suicide-related coping, depression, negative problem orientation and suicide-relevant cognitions.

Key findings regarding covariates are noteworthy. First, individuals who endorsed the pre-treatment measures with the most distress demonstrated the greatest improvement, which may indicate ceiling/floor effects for individuals who enter the program with low depression scores and high suicide related coping at the outset of treatment, or greater regression to the mean for those entering with greater distress. This finding is consistent with how Veterans have responded to Cognitive Behavioral Therapy for Suicide Prevention, another treatment offered in SP 2.0 (Betthauser et al., 2025). Results support the utility of PST-SP for, and importance of outreach to, populations endorsing greater distress via high depression and low suicide coping skills.

Second, relatively few covariates were significantly associated with outcomes over time, and PST-SP may be broadly beneficial across demographic and clinical groups. Though some covariates were related to initial levels of suicide-related coping and cognitions and depressive symptoms, associations with change throughout PST-SP were mostly minimal. However, it is worth noting that Hispanic ethnicity was related to greater decreases in depressive symptoms. While the broader absence of racial and ethnic differences in outcomes is consistent with previous research on PST for depression showing that diverse populations benefit from PST with no significant difference from White participants, the more significant reduction in depressive symptoms among Hispanics is also consistent with findings that problem-solving could be impacted by cultural factors and acculturative stress, which leaves room for improvement when provided support (Lee et al., 2018; Schmaling, 2019). Additionally, lower initial acute suicide risk was associated with greater improvement in depressive symptoms and negative problem orientation throughout PST-SP. Thus, other treatments may be more appropriate for individuals with higher acute suicide risk; this conclusion is consistent with general CPG recommendations around acute risk and treatment choices (VA/DoD, 2024). However, note that data on acute and chronic baseline suicide risk were available only for about half of the cohort—more research on the impact of acute risk and PST-SP effectiveness is necessary.

Hatcher and colleagues (2011) examined occurrences of self-harm following PST and found that 13.4% of patients who received PST self-harmed again. In the same study, 14.1% of those receiving usual care presented with self-harm; there was not a statistically significant difference between treatment arms. However, there was a significantly lower rate of self-harm for the PST arm among individuals with histories of repeated/multiple self-harm incidents prior to the study (13.5% in PST compared to 22.1% in usual care). Though these samples are not directly comparable due to differences in methods and constructs (i.e., self-harm may include non-suicidal, vs. SSDV), in the current PST-SP cohort, the rate of SSDV was similar to the rates found by Hatcher et al (2011), at 15.9% at 12 months. This supports a conclusion that PST-SP as delivered via telehealth in SP 2.0 leads to comparable rates of suicide-related behaviors as shown previously with PST. The comparison data presented above—10.1% with SSDV among non-SP 2.0 Veterans with SSDV within 12 months of being seen in general mental health—reflect slightly lower rates of SSDV. It is possible that this comparison group may represent a slightly lower-risk population, such that those at greater risk tend to be referred to SP 2.0; around half of the PST-SP cohort are at intermediate to high chronic suicide risk, and almost a third at intermediate to high acute risk. However, comparisons of risk in SP 2.0-referred Veterans to those not engaged in SP 2.0 have not been directly tested. The comparisons provided here provide context, but are not a substitute for controlled data from a randomized controlled trial, as they involve different cohorts, methods, etc.

Despite the clinical improvements among Veterans who received PST-SP, there were several limitations of the current project: (1) Our study used a retrospective, medical record review design to evaluate the association of PST-SP covariates with clinical outcomes for Veterans in “real-world” clinical settings within a complex national healthcare system, rather than evaluating PST-SP using a randomized controlled trial design to control for potential confounding and bias. Thus, conclusions regarding the definitive efficacy of PST-SP are not possible. Additionally, reflective of “real-world” implementation, non-completion of the full treatment was fairly common, with several completing as few as one or two sessions (Fig. 1). (2) Self-report questionnaires were not consistently administered and collected in a standardized fashion in the real-world implementation of PST-SP, leading to incomplete data sets; sample sizes for analyses of suicide-related coping, depressive symptoms, negative problem orientation, and suicide-relevant cognitions were much smaller than the total sample of Veterans who engaged in PST-SP. Certain measures (e.g., SCS-R) were more frequently administered than others and thus have larger sample sizes. Missingness may not have been at random; those who were not administered the questionnaires may systematically differ in some way from those who were. (3) Further, the questionnaire results are shared and discussed between the Veteran and therapist directly in session, contributing to the risk of social desirability impacting self-report of treatment outcomes.

## Conclusions

Results from this study show that PST-SP broadly leads to improvements in suicide-related coping, suicide-relevant cognitions, depressive symptoms, and problem orientation. The current project is an important first step in demonstrating the feasibility and effectiveness of PST-SP as a fully virtual clinical treatment for reducing suicide risk, and its positive outcomes on a high-risk group of Veterans. Further research is warranted to more definitively establish the efficacy and effectiveness of PST-SP and to further examine potential population differences in responses to PST-SP.

## Data Availability

Data include PHI from VA healthcare systems and are thus not publicly available.

## Code availability

Analyses were conducted using widely available software applications. Custom code is not available due to it describing backend components of VA data systems.
